# Molecular systematics of the genus *Troglophilus* (Rhaphidophoridae, Orthoptera) in Turkey: mitochondrial 16S rDNA evidences

**DOI:** 10.3897/zookeys.257.4133

**Published:** 2013-01-07

**Authors:** Mehmet Sait Taylan, Claudio Di Russo, Mauro Rampini, Valerio Ketmaier

**Affiliations:** 1 The Society of Anatolian Speleology Group (ASPEG), Serpil Sk., Yıldız Apt. 14/A, Kavacık, Beykoz, İstanbul, Turkey; 2 Dipartimento di Biologia e Biotecnologie “C. Darwin” (Zoologia), Università degli Studi di Roma “La Sapienza”, Viale dell’Università 32, 00185 Roma, Italy; 3 Unit of Evolutionary Biology/Sytematic Zoology, Institute of Biochemistry and Biology, University of Potsdam, Karl-Liebknecht Str. 24-25, Haus 26, D-14476, Potsdam, Germany

**Keywords:** *Troglophilus*, Rhaphidophoridae, Orthoptera, 16S rDNA, mitochondrial DNA, molecular systematics, cave crickets

## Abstract

This study focuses on the evolutionary relationships among Turkish species of the cave cricket genus *Troglophilus*.Fifteen populations were studied for sequence variation in a fragment (543 base pairs) of the mitochondrial DNA (mtDNA) 16S rDNA gene (16S) to reconstruct their phylogenetic relationships and biogeographic history. Genetic data retrieved three main clades and at least three divergent lineages that could not be attributed to any of the taxa known for the area. Molecular time estimates suggest that the diversification of the group took place between the Messinian and the Plio-Pleistocene.

## Introduction

Caves are traditionally considered as natural laboratories to understand evolutionary processes related to allopatric divergence because, similarly to remote oceanic islands, by their very nature greatly reduce or hamper gene flow among populations (Poulson and White 1969; Sbordoni 1982; Barr and Holsinger 1985; Sbordoni et al. 1987; Venanzetti et al. 1993; Di Russo et al. 1998). Here we present a case study based on populations and species of the cave crickets genus *Troglophilus* from Turkish caves. This genus belongs to family Rhaphidophoridae, which has a worldwide distribution and typically includes wingless crickets with a clear preference for dump environments, including natural and artificial caves. In the Northern hemisphere these crickets are essentially confined to natural and artificial caves. Overall 10 subfamilies have been recognized to date (Rentz 1991; Di Russo and Sbordoni 1998; Gorochov 2001; Otte 2000; Eades et al. 2011).

In the peri-Mediterranean area the family is represented by two genera only (*Dolichopoda* and *Troglophilus*) with a fairly overlapping Eastern-Mediterranean distribution. *Dolichopoda* (49 described species) is by far more species-rich than *Troglophilus* (17 described species). Until now, seven species of *Dolichopoda* (*Dolichopoda aranea* Bolivar, 1899, *Dolichopoda pusilla* Bolivar, 1899, *Dolichopoda euxina* Semenov, 1901, *Dolichopoda sbordonii* Di Russo & Rampini, 2006, *Dolichopoda lycia* (Galvagni, 2006), *Dolichopoda noctivaga* Di Russo & Rampini, 2007, *Dolichopoda sutini* Rampini & Taylan, 2012) and five species of *Troglophilus* (*Troglophilus escalerai* Bolivar, 1899, *Troglophilus gajaci* Us, 1974, *Troglophilus adamovici* Us, 1974, *Troglophilus bicakcii* Rampini & Di Russo, 2003, *Troglophilus tatyanae* Di Russo & Rampini, 2007) have been reported from Anatolian caves. As far as *Troglophilus* is concerned, the first species to be described from the area was *Troglophilus escalerai* (Jenidje-Kale cave) by Bolivar in 1889. After this early study, Us described *Troglophilus adamovici* (Isparta, Zindan cave) and *Troglophilus gajaci* (Mersin, Cennet cave) in 1974. About thirty years later Rampini and Di Russo (2003) identified the new taxon *Troglophilus bicakcii* (Derebucak, Bıçakçı Cave), while the description of *Troglophilus tatyanae* (Artvin, Kafkasor) was presented in Di Russo et al. (2007).

Of these two genera of cave crickets inhabiting the peri-Mediterranean area, *Dolichopoda* has received comparatively more scientific attention than *Troglophilus*. Both genera have been the object of a number of studies based on a variety of molecular markers. Nowadays for *Dolichopoda* we have a very detailed knowledge from the population level (with special emphasis on those species inhabiting the Italian peninsula) up to the phylogenetic relationships among the vast majority of taxa ascribed to the genus (Allegrucci et al. 2011 and references therein). Genetic studies conducted on *Troglophilus* have considered the Italian, Balkan, insular Greek and Anatolian species (Sbordoni et al. 1981; Cobolli et al. 1999; Ketmaier et al. 2000, 2004, 2010) but a well-resolved phylogeny of the genus is still awaited.

Cobolli et al. (1999) used allozymic markers to disentangle relationships among Anatolian species of *Troglophilus* from the Taurus Mountains between Isparta and Adana towns. The study revealed four distinct gene pools including the three species *Troglophilus adamovici*, *Troglophilus escalerai* and *Troglophilus gajaci* plus a genetically differentiated form that was later described as the new species *Troglophilus bicakcii* by Rampini and Di Russo (2003). That was a preliminary study; indeed only a limited number of populations were screened genetically and the markers employed (allozymes) notoriously reveal just a limited fraction of the total genetic variation. More recently, Kaya et al. (2012) presented a phylogeographic hypothesis for the Anatolian *Troglophilus*; the samplings in that and in the current study largely overlap but those authors did not include *Troglophilus escalerai* in their analyses. Markers differed between the studies; Kaya et al. (2012) sequenced fragments of the mitochondrial Cytochrome Oxidase I and II genes and the nuclear region spanning the Internal Transcribed Spacers 1 and 2. Anatolian representatives clustered in a monophyletic group of Miocene origin; divergence within the Anatolian clade occurred through the Plio-Pleistocene but earlier than the last four glacial periods of the late Pleistocene.

**Figure 1.**
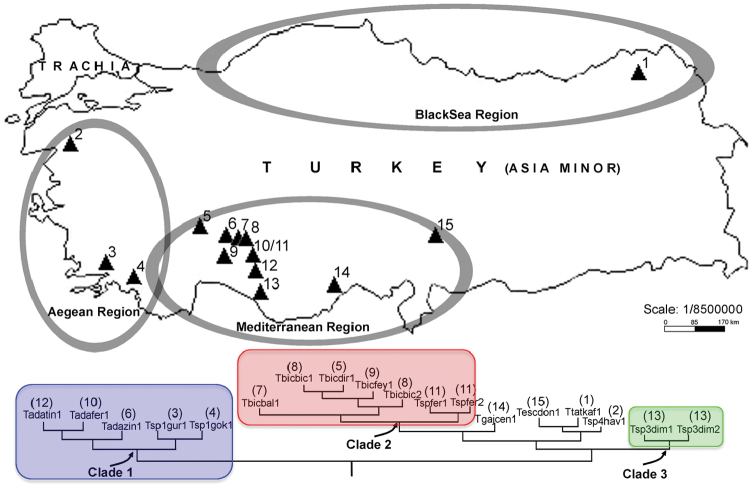
Geographic position of the fifteen caves were we sampled the *Troglophilus* populations analyzed in the study. Numbers correspond to those in Table 1. The lower half of the figure depicts the phylogeography of *Troglophilus* in Turkey (for details see Discussion); colors of clades match those in Figure 2.

For this study, we explored 71 caves from the Black Sea, Aegean, Mediterranean and inland areas of Turkey and found and collected cave crickets belonging to the genus *Troglophilus* from 15 of them (Figure 1; Table 1). We included in the study all the five known Turkish species of *Troglophilus*, including *Troglophilus escalerai* that was not analyzed in Kaya et al. (2012). For some species we were able to collect multiple populations (Table 1). Samples were screened for sequence variation at the mitochondrial DNA (mtDNA) 16S rDNA gene (16S). The gene is known to be informative at the closely related species level in insects (Simon et al. 1994). The aims of this study are to reconstruct the evolutionary relationships among the Turkish *Troglophilus* species, to use genetic data to clarify the systematics of the group in the area and, ultimately, to identify the evolutionary trajectories it followed in the course of its diversification. The newly acquired data will be discussed in light of the results obtained by Cobolli et al. (1999) and Kaya et al. (2012). Patterns of relationships within *Troglophilus* will be finally compared to those presented in Allegrucci et al. (2011) for *Dolichopoda* for the same area to search for eventually overlapping patterns in two groups with similar ecologies.

**Table 1.** Species list and details of the sampling localities of Turkish *Troglophilus* populations and species. Numbers in the first column match those in Figure 1.

No	Species	Cave name	Locality	N (north), E (east)	Date	Altitude (m a.s.l.)
**Black Sea Region**							
1	*Troglophilus tatyanae*	Epigian forest	Artvin, Kafkasor	41.098, 41.475	29–30/06/2000	1300
**Aegean Region**							
2	*Troglophilus* sp .4	Havran cave	Balıkesir, Havran	39.34499, 27.10336	01/11/2008	115
3	*Troglophilus* sp .1	Gökçeler cave	Muğla, Milas	37.11378, 27.45982	25/11/2008	120
4	*Troglophilus* sp .1	Güroluk cave	Muğla, Fethiye	36.47564, 28.58646	26/06/2008	450
**Mediterranean and Central Anatolia Region**							
5	*Troglophilus adamovici*	Zindan cave	Isparta, Aksu	37.48424, 31.05060	03/05/2009	1286
6	*Troglophilus bicakcii*	Direkliin cave	Konya, Beyşehir	37.35548, 31.28549	02/07/2008	1209
7	*Troglophilus bicakcii*	Bıçakçı cave	Konya, Derebucak	37.23648, 31.32166	23/08/2009	1372
8	*Troglophilus bicakcii*	Balatini cave	Konya, Derebucak	37.21706, 31.35060	22/08/2009	1379
9	*Troglophilus bicakcii*	Feyzullah cave	Konya, Derebucak	37.15771, 31.27314	22/08/2009	1508
10	*Troglophilus adamovici*	Ferzene cave	Konya, Seydişehir	37.22854, 31.50071	24/08/2009	1390
11	*Troglophilus* sp .2	Ferzene cave	Konya, Seydişehir	37.22854, 31.50071	24/08/2009	1390
12	*Troglophilus adamovici*	Tınaztepe cave	Konya, Seydişehir	37.14855, 31.35692	24/08/2009	1461
13	*Troglophilus* sp .3	Dim cave	Antalya, Alanya	36.32405, 32.06549	30/08/2009	232
14	*Troglophilus gajaci*	Cennet cave	Içel, Silifke	36.27120, 34.06383	05/06/2009	135
15	*Troglophilus escalerai*	Döngel cave	Maraş, Narliseki	37.51557, 36.38476	06/06/2009	647

## Methods

### Sampling and studying methods

Ten caves have been checked for each region in Turkey (Mediterranean, Central Anatolian, Aegean and Black Sea region) to collect cave crickets and fifteen sampled populations belonged to the genus *Troglophilus*; of these eleven were in the Mediterranean and Anatolian region, three in the Aegean region and one in the Black Sea region (Figure 1). All the known five Turkish species (*Troglophilus escalerai*, *Troglophilus gajaci*, *Troglophilus adamovici*, *Troglophilus bicakcii*, *Troglophilus tatyanae*) and four new taxa/populations from Muğla, Alanya, Seydişehir and Balıkesir provinces (see Table 1, Figure 1) were included in this study. The latter four taxa are hereto considered as non-described species because it was not possible to attribute them on morphological grounds to any of the *Troglophilus* species known for the area. Specimens were collected between 2008 and 2009 by hands searching on walls and grounds of caves through the day. Morphological identification of specimens was performed using a stereomicroscope Leica MZ 12.5 equipped with a “camera lucida” and photo camera. Specimens were preserved in absolute ethyl alcohol at AUZM (Akdeniz University Zoology Museum, Antalya, Turkey).

### DNA isolation, PCR (Polymerase Chain Reaction) and DNA sequencing

Genomic DNA was extracted from the hind femoral muscle using I-genomic CTB DNA Extraction Mini Kit (type G protocol for Insect, Cat. No 17341, Macrogen Inc.). A 532-535 base pair (bp) fragment of the mitochondrial 16S rDNA gene was amplified through the Polymerase Chain Reaction (PCR) from each individual samples. The primers used were ER232 (5’-CGCCTGTTTAACAAAAACAT-3’) and ER233 (5’-CCGGTCTGAACTCAG ATGACTG-3’) (Simon et al. 1994). PCR amplifications were performed with a Bio-Rad PTC0220 cycler (Macrogen Inc.) in a 50 µl reaction volume containing genomic DNA (50-100 ng), 25 mM dNTP, 10 µl Band Doctor (5x) 5 µl Buffer (10x), 2 µl (10 pmol/ µl) of each primer, 0.3 µl Ex-Taq (5U/ µl) and distiller water. The PCR conditions were as follows: 95 ^o^C for 5 minutes, followed by 39 cycles of denaturation at 95 ^o^C for 30 s, annealing of primers at 53 ^o^C for 30 s, elongation at 72 ^o^C for 1 min and one final extension step at 72 ^o^C for 5 min. PCR products were purified using the QIAquick PCR Purification Kit (Qiagen); in some circumstances PCR products were excised from gel and purified with the QIAquick Gel Extraction Kit (Qiagen). Sequencing was carried out on an ABI 3730XL sequencer in both directions and with the same primer pair used for PCRs. Sequences data were edited and compiled using Codoncode Aligner (Codoncode Corporation MA, USA version 2.0.2).

### Phylogenetic and divergence time analyses

Sequences were aligned in ClustalX (Thompson et al. 1997) with default parameters. Aligned sequences were analyzed phylogenetically by maximum parsimony (MP; heuristic searches, ACCTRAN character-state optimization, 100 random stepwise additions, TBR branch-swapping algorithm) (Farris 1970) and Bayesian methods (Rannala and Yang 1996; Mau and Newton 1997; Larget and Simon 1999; Mau et al. 1999; Huelsenbeck et al. 2000). MP analyses were performed using paup* 4.0b10 (Swofford 2003); Bayesian analysis was carried out using MrBayes 3.1 (Ronquist and Huelsenbeck 2003). MP searches were run giving equal weight to all substitutions. We determined the best model of DNA substitutions fitting our data using JMODELTEST (Posada 2008); the chosen model was then used for the Bayesian analyses allowing site-specific rate variation. MrBayes was run for 2 million generations with a sampling frequency of 100 generations. We ran one cold and three heated Markov chains. From the 20000 trees found, we discarded the first 10% (“burn-in”) in order to include only trees for which convergence of the Markov chain had been reached; the posterior probabilities were estimated only for those generations sampled after the burn-in. The remaining 18000 trees were used to construct a 50% majority rule consensus tree using paup* 4.0b10. The robustness of the MP hypotheses was tested by 1000 bootstrap replicates (Felsenstein 1985). In addition, we sequenced a single individual of *Dolichopoda geniculata* from Valmarino cave (Latium, Central Italy); the species belongs to the only other Rhaphidophoridae genus present in the Mediterranean area and was used as the outgroup for all phylogenetic searches. We calculated Maximum Likelihood (ML) genetic distances among the main lineages retrieved from the phylogenetic searches using the settings yielded by JMODELTEST.

Divergence times were calculated in a Bayesian MCMC framework by using Beast 1.4.6 (Drummond and Rambaut 2007). We adopted a model of uncorrelated but log-normally distributed rates of molecular evolution (Drummond et al. 2006). Neither fossil evidence nor geological events for the species analyzed here were available to calibrate our phylogeny. Consequently, we took advantage of the 16S substitution rate of 0.7% per lineage per million years estimated by Allegrucci et al. (2011) for the cave cricket genus *Dolichopoda* to date age of divergence among haplotypes. We used a Yule prior on rates of evolution because this more accurately resembles phylogenetic processes at the species level. We adopted the same GTR+ G model as in the ML and Bayesian searches. We ran five independent analyses of 50,000,000 generations each; the corresponding outputs were analyzed using Tracer 1.4, TreeAnnotator 1.4.6 and FigTree 1.0 (Drummond and Rambaut 2007). A Mantel test (Mantel 1967), considering all in-group taxa, was carried out to test for a possible correlation between genetic and geographic distances.

## Results

### Sequence variation

The 16S alignment consisted of 543 nucleotidic positions. Sequences were obtained for each individual and a total of 38 samples belonging to 15 populations were analyzed and 18 different haplotypes found. Sequences of these unique haplotypes have been deposited in GenBank under the Accession N. JX968473-JX968490. Table 2 shows the absolute frequency of these 18 haplotypes in the different populations included in the study. In the final alignment 123 sites were variable and 53 were parsimony informative. The transition/transversion (ti/tv) ratio ranged from 1.7 to 2.2. Ti values accounted for about 62% or 69% of all substitutions when the outgroup was alternatively included or excluded. Divergence in the 16S rDNA gene ranged from 1.1% to 13.1% at the ingroup level (16.1% with the outgroup included).

**Table 2.** *Troglophilus* species included in this study, the names of the sampling locations, their sample size per locality (*N*), number of haplotypes, the codes of the haplotypes as they appear in Figure 1 and 2.

**Species**	**Population**	**Locality**	**N**	**Haplotype number**	**Haplotype code**
**Black Sea Region**					
*Troglophilus tatyanae*	Kafkasor	Artvin	1	1	Ttat-kaf1
**Aegean Region**					
*Troglophilus* sp .1	Güroluk cave	Muğla, Fethiye	3	1	Tsp1-gur1
	Gökçeler cave	Muğla, Milas	1	1	Tsp1-gok1
*Troglophilus* sp .4	Havran Cave	Balıkesir, Havran	3	1	Tsp4-hav1
**Mediterranean and Central Anatolia region**					
*Troglophilus escalerai*	Döngel cave	K.Maraş, Döngel	3	1	Tesc-don1
*Troglophilus gajaci*	Cennet cave	İçel, Silifke	5	1	Tgaj-cen1
*Troglophilus adamovici*	Zindan cave	Isparta, Aksu	4	1	Tada-zin1
	Tınaztepe cave	Konya, Seydişehir	2	1	Tada-tin1
	Ferzene cave	Konya, Seydişehir	1	1	Tada-fer1
*Troglophilus bicakcii*	Bıçakçı cave	Konya, Derebucak	2	2	Tbic-bic1, Tbic-bic2
	Direkliin cave	Konya, Beyşehir	2	1	Tbic-dir1
	Feyzullah cave	Konya, Derebucak	2	1	Tbic-fey1
	Balatini cave	Konya, Derebucak	2	1	Tbic-bal1
*Troglophilus* sp .2	Ferzene cave	Konya, Seydişehir	2	2	Tsp2-fer1, Tsp2-fer2
*Troglophilus* sp .3	Dim Cave	Antalya, Alanya	5	2	Tsp3-dim1,Tsp3-dim2

### Phylogenetic analyses and divergence times

**Figure 2.**
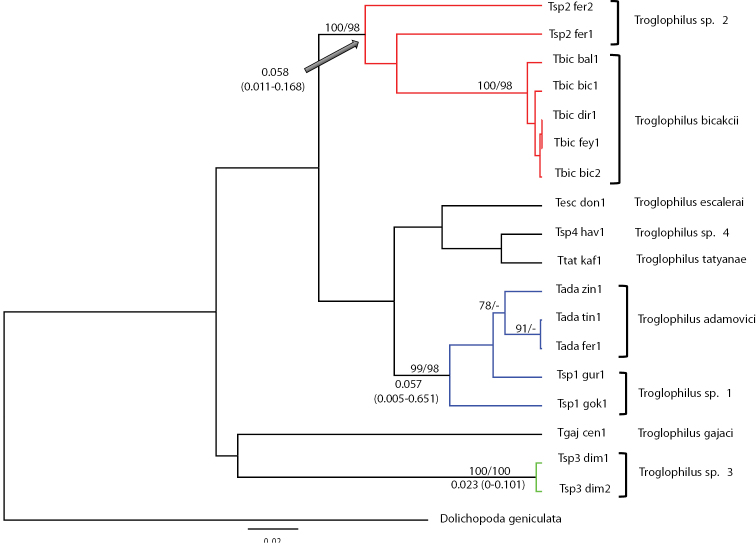
Bayesian phylogram among *Troglophilus* haplotypes from Turkey. Haplotype codes match those in Table 2. Numbers at nodes are statistical supports for the Bayesian and MP searches (first and second value, respectively); only values ≥ 75% are reported. The three supported clusters are described in the text are highlighted here in blue (clade 1), red (clade 2) and green (clade 3). Bold values are node ages (in Myr %) as obtained by the BEAST analyses; 95% HPD intervals are shown in parentheses.

Figure 2 shows the Bayesian phylogram based on the GTR + G (gamma distribution shape parameter a = 0.188) model chosen by JMODELTEST as the one best fitting our data and summarizes the results of the other phylogenetic methods employed in the study. Bayesian and MP searches were all largely congruent with one another. MP searches yielded three equally parsimonious trees with length (L) = 193 steps, homoplasy index (HI) = 0.249, consistency index (CI) = 0.751, retention index (RI) = 0.780. All analyses consistently recovered three well-supported clades, whose geographic distribution is shown in Figure 1.

Clade 1 includes *Troglophilus adamovici* and *Troglophilus* sp. 1 populations, which are distributed in the Northern Mediterranean region (Isparta) through the western Taurus Mountain, Southern Central Anatolian regions with a Mediterranean climate and Southern Aegean region (Muğla, Fethiye, Milas). Clade 2 contains *Troglophilus bicakci* and *Troglophilus* sp. 2 populations, which are distributed in the Southern Central Anatolian region through Kembos Polye and Konya, Seydişehir, Derebucak and Beyşehir Provinces. This clade overlaps with Clade 1 in the Seydişehir Province (Ferzene cave). Clade 3 comprises *Troglophilus* sp. 3 population only and it is geographically restricted to the Antalya area (Alanya, Dim cave). The cave is located near the Dim River in the Southern Mediterranean Region.

Average GTR + G distance between Clade 1 and 2 is 0.063 ± 0.025, between Clade 2 and 3 is 0.058 ± 0.021 and between Clade 1 and 3 is 0.050 ± 0.005. Time estimates retrieved from the Bayesian MCMC analyses for the three main clades are illustrated in Figure 2. In all cases 95% credible intervals for node age estimates overlapped. The data did not conform to a clock-like behavior, the coefficient of variation being 0.87 (95% High Posterior Density, HPD: 0.393-1.435; ESS: 1214.24). Parent and daughter branches showed no co-variation, the mean covariance being -5.83^-2^ (HPD: -0.321-0.237; ESS: 7191.33). The 95% High Posterior Density spans zero; this implies that branches with fast and slow rates are next to each other in the phylogenetic tree. There is thus no evidence of autocorrelation of rates in the tree. Ages of Clades 1, 2, and 3 ranges between 5.8 and 2.3 million years; the lack of a clear calibration point resulted in a chronogram with relatively ample confidence intervals (Figure 2).

Results of the Mantel test (Mantel 1967), performed to explore a possible correlation between geographic and genetic distance in all studied taxa, suggested there was no correlation between genetic and geographic distances (r= -0.01, p value (two-tailed) = 0.881).

## Discussion

### Molecular systematics

The genetic data confirmed the validity of the already described species, with conspecific populations firmly forming monophyletic clusters. On the other hand, four deeply genetically divergent lineages (*Troglophilus* sp. 1, 2, 3 and 4) could not be attributed to any of the previously described species and could hence represent new taxa. The mean GTR + G genetic distance between the described *Troglophilus* species included in our study (Bolivar 1899; Us 1974; Rampini and Di Russo 2003; Di Russo et al. 2007) ranges from 0.028 to 0.065 ± 0.008. The four new taxa (*Troglophilus* sp. 1, 2, 3 and 4) diverge from all the described species for a GTR + G distance range comprised between 0.023-0.132 ± 0.026. Hence, these four new lineages are genetically as divergent as the formally described species are, and in some cases even more.. In addition, they also show morphological differences in the shape of the ovipositor, which is one of the most important discriminating characters traditionally used for taxonomic purposes in *Troglophilus* (Taylan et al. 2011).

Cobolli et al. (1999), by using allozymes revealed four distinct gene pools among Anatolian species of *Troglophilus* from the Taurus Mountains between Isparta and Adana provinces. These corresponded to *Troglophilus adamovici*, *Troglophilus gajaci* and two lineages formally not described yet genetically differentiated. One of those lineages was later described as the new species *Troglophilus bicakcii* by Rampini and Di Russo (2003) (from the Balatini cave), while the second lineage is the *Troglophilus* sp. 2 from the Ferzene cave included in the present study. *Troglophilus* sp. 1, 3 and 4 were not reported in Cobolli et al. (1999). It is worth noting that *Troglophilus* sp. 2 is syntopic with *Troglophilus adamovici* (Taylan et al. 2011).

Overall, we could distinguish three main clades; all received strong support in our phylogenetic analyses (Figures 1 and 2). Clade 1 includes *Troglophilus adamovici* and the new species *Troglophilus* sp. 1 distributed in the Isparta, Konya and Izmir provinces. Clade 2 comprises *Troglophilus bicakcii* and the new species *Troglophilus* sp. 2 (from Ferzene cave) both from the Konya province, while Clade 3 includes *Troglophilus* sp. 3 population distributed in the Dim Cave in Antalya. The phylogenetic placement of *Troglophilus gajaci*, *Troglophilus escalerai*, *Troglophilus tatyanae*, and *Troglophilus* sp. 4 could not be resolved by the data and remains controversial. Kaya et al. (2012), by using a combination of mitochondrial and nuclear genes, found good support for *Troglophilus gajaci* basal to a group of non-described forms, including a population corresponding to *Troglophilus* sp. 3 in our study. The placement of *Troglophilus tatyanae* is not resolved in either study, while Kaya et al. (2012) consistently retrieved a sister-species relationship for *Troglophilus adamovici* and *Troglophilus bicakcii*. Those authors did not analyze *Troglophilus escalerai*. It is evident that these discrepancies could be reconciled only by maximizing the overlap of both species and markers. Another point that shouldn’t be overlooked is that a phylogenetic hypothesis for the whole genus *Troglophilus* is still missing. A study based on a multi-gene approach and aimed at producing such a hypothesis is in progress, which will likely shed light on the questions left open by this and previous studies.

### Phylogeography

The Mantel test (Mantel 1967) shows that there is no correlation between genetic and geographic distances; hence genetic divergence is not function of the geographic distance separating the different caves. Considering the high level of genetic divergence found among our populations, we conclude that mitochondrial gene flow among these populations broke off completely sometimes in the past. This scenario is similar to what observed in subterranean diving beetles in isolated aquifers in Australia (Leijs et al. 2012), but, quite unexpectedly, it is different from that retrieved for the only other Mediterranean cave crickets (genus *Dolichopoda*). As a matter of fact,Allegrucci et al. (2005) and Taylan et al. (unpublished data) found strong evidence supporting isolation by distance pattern in *Dolichopoda*. The difference in the genetic structure between *Troglophilus* and *Dolichopoda* could be due to a higher tendency for the latter to maintain gene flow among caves. On the other hand, it shouldn’t be overlooked that our sampling across Turkey is rather sparse and isolation by distance could fail to emerge from the data just because we missed too many intervening locations in our sampling. Finally, our study is based on a single marker with moderate evolving rates. On a more local scale, with a denser sampling and a multi-gene approach, isolation by distance was found in *Troglophilus cavicola* in Northern Italy (Ketmaier et al. 2004), suggesting that the result of the present study could be either sampling or marker-biased.

An additional point of interest of this study is the confirmation of the results of Cobolli et al. (1999) supporting the syntopic occurrence of two genetically divergent lineages in the Ferzene cave (*Troglophilus adamovici* and *Troglophilus* sp. 2). This pattern suggests a secondary contact of these lineages after allopatric divergence, a phenomenon reported multiple times in cave dwelling-organisms (Sbordoni et al. 2000; Niemiller et al. 2008; Raşit et al. 2008). As a matter of fact, Cobolli et al. (1999) found nine allozymic loci fixed for alternative alleles with no heterozygotes in the large number of samples (147) used for that study. We could observe no sign of mitochondrial DNA introgression in the few samples we analyzed for the study. Based on previous allozymic data but keeping in mind our limitations in terms of sample size and markers, we would tentatively conclude that these two syntopically occurring lineages are reproductively isolated. It is evident that a multi-gene approach, based on both mitochondrial and nuclear fast evolving markers, is necessary to properly address the issue. It is nonetheless worth noting that the syntopic co-occurrence of closely related, non-intermixing lineages would imply a differential exploitation of resources to avoid competition. It is reasonable to hypothesize that these two divergent lineages have acquired (slightly) different ecological niches, a point that would be interesting to address with an *ad-hoc* designed study.

The estimated divergence times range from the Messinian to the Plio-Pleistocene (Figure 2). The oldest estimated divergence times are around 5.8 Ma (Messinian) and coincide with the last period of the uplifting the Anatolian Plateau, which arose 5-10 Ma as a consequence of the northward movement of the Arabian Plate (Qennell 1984; Steininger and Rögl 1984). The Messinian was a time of high rainfall and high sediment yields rates (Zeit Wet Phase, Griffin 1999). This phase, characterized by a humid climate, might have favored regional dispersal. The fact that our divergence times within Clades 1 and 2 are near the end of this wet phase suggests that the transition towards the drier Messinian climate was responsible for the splits. Cave crickets (and cave organisms in general) (Carchini et al. 1991; Taylan et al. 2011) cannot withstand epigean dry conditions; we envision a scenario where these crickets were forced to seek refuge in the subterranean environment during the Messinian and started diverging in allopatry. These estimates are in remarkable agreement with those obtained for the genus *Dolichopoda* in the Eastern Mediterranean area (Allegrucci et al. 2009).

The estimated divergence time for *Troglophilus* sp. 3 is more recent (2.3 Ma), dating to the Plio-Pleistocene, which was characterized by alternating dry/cold and warm/humid phases. The climatic fluctuations during the Plio-Pleistocene likely led to ecological fragmentation with subsequent genetic isolation and speciation in the area. This hypothesis is also supported by the results from the *Dolichopoda* species, whose radiation also appears to have followed the climatic changes of the Plio-Pleistocene (Allegrucci et al. 2005, 2009).

Since the syntopic *Troglophilus adamovici* and *Troglophilus* sp. 2 in the Ferzene cave do not interbreed, their secondary contact must have taken place after the diversification within Clades 1 and 2, certainly more recently than the Messinian. Even though we are not in the position to date when the secondary contact actually happened, we suspected that this was favored by one of the many warm and humid climatic phases of the Quaternary, which allegedly promoted epigean dispersal among lineages that had been previously confined to caves.

Our time estimates for the splitting events within the Anatolian representatives of *Troglophilus* are in agreement with those reported in Kaya et al. (2012). This concordance is even more remarkable considering the differences between the two studies in terms of sampled taxa, markers employed and (at least partially) phylogenetic relationships retrieved (see the molecular systematics section). Also those authors identified the climate changes of the Plio-Pleistocene as the cause that triggered divergence among Anatolian *Troglophilus*.

Finally, it should not be overlooked that this study is limited to the Turkish area and is based on a single mitochondrial marker. To place these results in a broader perspective and to understand in details the evolutionary trajectories followed by the genus, we need to expand our sampling by covering its whole distribution range and by combining multiple mitochondrial and nuclear loci. To these aims our ongoing research activity is currently devoted.
